# Predicting the “usefulness” of 5-ALA-derived tumor fluorescence for fluorescence-guided resections in pediatric brain tumors: a European survey

**DOI:** 10.1007/s00701-014-2234-2

**Published:** 2014-09-24

**Authors:** Walter Stummer, Floriano Rodrigues, Philippe Schucht, Matthias Preuss, Dorothee Wiewrodt, Ulf Nestler, Marco Stein, José Manuel Cabezudo Artero, Nunzio Platania, Jane Skjøth-Rasmussen, Alessandro Della Puppa, John Caird, Søren Cortnum, Sam Eljamel, Christian Ewald, Laura González-García, Andrew J Martin, Ante Melada, Aurelia Peraud, Angela Brentrup, Thomas Santarius, Hans Herbert Steiner, For the European ALA Pediatric Brain Tumor Study Group

**Affiliations:** 1 Department of Neurosurgery, Universitätsklinikum Münster, Albert-Schweitzer-Campus 1, 48149 Münster, Germany; 2 medac GmbH, Theaterstr. 6, 22880 Wedel, Germany; 3 Neurosurgical Department, Freiburgstr. 2, 3010 Bern, Switzerland; 4 Pediatric Neurosurgery, Department of Neurosurgery, University Hospital Leipzig, Liebigstr. 20, 4103 Leipzig, Germany; 5 Department of Neurosurgery, Justus-Liebig University Giessen, Klinikstr. 33, 35390 Giessen, Germany; 6 Complejo Hospitalario Universitario Infanta Cristina, Avenida de Elvas, 6010 Badajoz, Spain; 7 Neurosurgical Department, A.O.U. Policlinico - Vittorio Emanuele, Catania, Via Santa Sofia 78, 95123 Catania, Italy; 8 Neurosurgical Department, Rigshospitalet, Blegdamsvej 9, 2100 Copenhagen Ø, Denmark; 9 Department of Neurosurgery, University Hospital of Padova, Via Giustiniani 2, 35128 Padova, Italy; 10 Beaumont Hospital, Beaumont Road, Dublin 9, Ireland; 11 Aalborg Universityhospital, 9000 Aalborg, Denmark; 12 Ninewells Hospital and Medical School, Minewells Avenue, DD1 9SY Dundee, Scotland UK; 13 Department of Neurosurgery, Jena University Hospital, Erlanger Allee 101, 7747 Jena, Germany; 14 Carlos Haya University Hospital, Carlos Haya Street, 29010 Málaga, Spain; 15 Department of Neurosurgery, Atkinson Morley Wing, St George’s Hospital, SW17 0QT London, UK; 16 Department of Neurosurgery, UHC Zagreb, Kišpaticeva 12, 10 000 Zagreb, Croatia; 17 Department of Neurosurgery, Klinikum Grosshadern, Ludwig-Maximilians-University, Marchioninistr. 15, 81377 Munich, Germany; 18 Department of Neurosurgery, Addenbrooke’s Hospital, Hills Road, CB2 0QQ Cambridge, UK; 19 Department of Neurosurgery, General Hospital Nuremberg, Breslauerstr. 201, 90471 Nuremberg, Germany

**Keywords:** 5-ALA, Gliolan, Pediatric brain tumor, Medulloblastoma, Ependymoma, Glioblastoma, Anaplastic astrocytoma, Fluorescence-guided resection

## Abstract

**Background:**

Five-aminolevulinic acid (Gliolan, medac, Wedel, Germany, 5-ALA) is approved for fluorescence-guided resections of adult malignant gliomas. Case reports indicate that 5-ALA can be used for children, yet no prospective study has been conducted as of yet. As a basis for a study, we conducted a survey among certified European Gliolan users to collect data on their experiences with children.

**Methods:**

Information on patient characteristics, MRI characteristics of tumors, histology, fluorescence qualities, and outcomes were requested. Surgeons were further asked to indicate whether fluorescence was “useful”, i.e., leading to changes in surgical strategy or identification of residual tumor. Recursive partitioning analysis (RPA) was used for defining cohorts with high or low likelihoods for useful fluorescence.

**Results:**

Data on 78 patients <18 years of age were submitted by 20 centers. Fluorescence was found useful in 12 of 14 glioblastomas (85 %), four of five anaplastic astrocytomas (60 %), and eight of ten ependymomas grades II and III (80 %). Fluorescence was found inconsistently useful in PNETs (three of seven; 43 %), gangliogliomas (two of five; 40 %), medulloblastomas (two of eight, 25 %) and pilocytic astrocytomas (two of 13; 15 %). RPA of pre-operative factors showed tumors with supratentorial location, strong contrast enhancement and first operation to have a likelihood of useful fluorescence of 64.3 %, as opposed to infratentorial tumors with first surgery (23.1 %).

**Conclusions:**

Our survey demonstrates 5-ALA as being used in pediatric brain tumors. 5-ALA may be especially useful for contrast-enhancing supratentorial tumors. These data indicate controlled studies to be necessary and also provide a basis for planning such a study.

## Introduction

Five-aminolevulinic acid (5-ALA, Gliolan) is approved for fluorescence-guided resections of malignant gliomas in Europe and a number of countries abroad. Approval of 5-ALA was based on a randomized study in adults and is now commonly used [1–10]. ALA induces the accumulation of fluorescent protoporphyrin IX (PpIX) in malignant gliomas via the heme biosynthesis pathway. This fluorochrome can be visualized intra-operatively and is useful for the identification of residual tumor. Since its approval in 2007, numerous reports have appeared also describing possible uses of 5-ALA in tumors other than gliomas, such as meningiomas, metastases, and others [11–13].

In many pediatric brain tumors, safe maximal resections have also been linked to prognosis [14–20], and it has always been of interest whether 5-ALA would be useful in this patient population. However, the histologist encountered in typical intra-axial contrast-enhancing tumors in children are much more varied than in adults. Apart from the malignant gliomas, in which 5-ALA has an established track record in adults, pediatric tumors include primitive neuroectodermal tumors, ependymomas, pilocytic astrocytomas, medulloblastomas, and others. Little is known about the extent of PpIX accumulation and fluorescence after 5-ALA administration in these childhood tumors and whether this fluorescence is actually useful for surgery, thus justifying any risks involved in 5-ALA application. No controlled studies have been performed so far. Two case reports describe useful fluorescence in a pleomorphic astrocytoma [21], the other in a medulloblastoma [22]. In addition, Preuss et al. [23] published a compilation of 18 children operated on with Gliolan in a compassionate-use setting compiled from three neurosurgical centers in Germany and France and covering a variety of pediatric brain tumor pathologies. Results were equivocal as only eight of 14 tumors displayed fluorescence, which was in turn considered to be “useful” in only seven children (three glioblastomas, one pleomorphic neuroectodermal tumor, one ependymoma III, one anaplastic astrocytoma, and one pilocytic astrocytoma). In a further small series of three case reports, useful fluorescence was found in two glioblastoma, but not in a case of medulloblastoma [24], underlining ambiguous findings. In addition, more recently, another series was published, this time discussing the use of 5-ALA in 16 children operated on at a single center outside the framework of a controlled clinical trial and with an undefined source of 5-ALA [25]. The youngest child in this series was 1 year old. Again, a variety of different pediatric tumors was observed with 5-ALA apparently inducing fluorescence in only three of 16 children (one anaplastic astrocytoma, one pilocytic astrocytoma, one glioblastoma). The question of whether this fluorescence was truly helpful was not addressed. On the other hand, distinct increases in liver enzymes in response to 5-ALA were observed, such as an increase of alanine aminotransferase (ALT). This was more evident more in younger children [25].

Especially this last series cautions that there is uncontrolled use of 5-ALA outside of clinical trials in children, despite possible side-effects and potential limitations due to the many different brain tumor types that will potentially be treated.

These observations strongly underline the necessity for a controlled clinical trial of Gliolan in children with an emphasis on safety. However, since all results so far were rather ambiguous, it appeared wise to collect further data before planning such a trial. For this reason, certified Gliolan users throughout Europe were asked to submit their anonymous data on children operated in their centers in an off-label setting. The collected information was intended to create a larger database with the aim of identifying those children pre-operatively which might benefit from 5-ALA, in order to design a future controlled study.

## Methods

This retrospective survey on children and juveniles <18 years of age was based on a structured questionnaire that was distributed to Gliolan users throughout Europe who participate in the Gliolan risk management program mandated by the European Medicines Agency (EMA). This questionnaire required the following information:

- Patient characteristics: age, sex, weight, Karnofsky Performance Score (KPS)
- Preoperative symptoms (nausea, vomiting, seizures, ataxia, paresis, visual symptoms, other)
- Dosing information (dose, time of application prior to anesthesia)
- Preoperative magnetic resonance imaging (MRI) data
  - Location (intra- or extra-axial, supra- or infratentorial)
  - Degree of contrast enhancement (strong, weak/patchy, none)
- Recurrence status (yes or no)
- Preoperative symptoms
- Final histology
- Quality of fluorescence (strong, weak/patchy, none)
- “Usefulness” of 5-ALA, defined as a change in surgical strategy or identification of residual tumor based on 5-ALA fluorescence
- Resection status
- Adverse events or lack thereof

Twenty centers provided their data. No source data verification was performed for this study.

As all children were treated in an off-label setting and this was a retrospective cohort study, ethical committee approval was not acquired from any of the participants prior to off-label administration of Gliolan. Contributing surgeons indicate having obtained informed consent in all cases. Since all participating physicians were certified in the context of the European Risk Management Plan, experience in each center was high and patients were generally treated in accordance with the general principles recommended for the use in adults, i.e., application of 20 mg/kg body weight orally and post-operative light protection for 24 h.

Data on children published in the three-center compilation by Preuss et al. [23] were included in this investigation using the structured questionnaire.

### Statistics

Univariate and multivariate logistic regression analyses were performed for testing interrelationships between factors. The Chi-squared test was used for testing contingency tables of nominal data and the *t* test for comparing numerical data. Recursive partitioning analysis (RPA) was used for defining subgroups with/without the likelihood of having “useful” fluorescence. All statistical analyses, including RPA analysis, were performed using JMP statistics software (version 11.1.1).

## Results

**Fig. 1:**
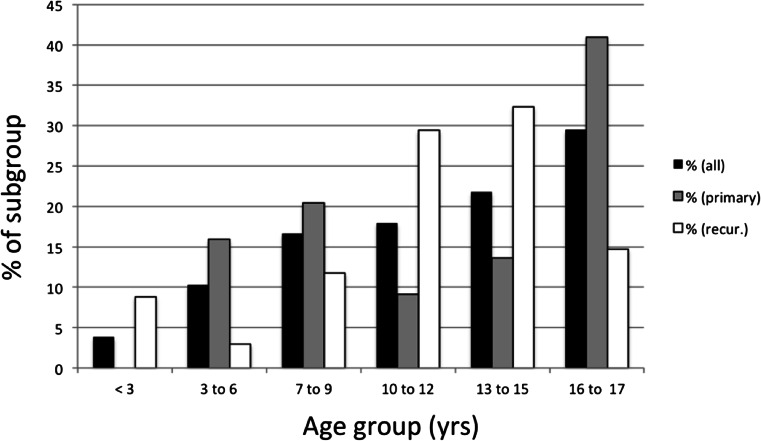
Distribution of ages for the complete cohort and stratified by recurrence status

Children’s demographic characteristics are given in Table 1. Figure 1 details the distribution of the children’s ages. The median age was 13, the median KPS was 90, and the median weight 42.4 kg. The most common preoperative symptom was “headache” (30/78; 38.4 %), followed by “nausea/vomiting” (20/78, 25.6 %), visual symptoms (13/78, 16.7 %), “paresis” (9/78, 11.5 %), and ataxia (8/78, 10.2 %). “Other symptoms” included changes in personality, somnolence (*n* = 2), drowsiness, loss of fine movements of hand, nystagmus, abducens nerve paresis (*n* = 2), neck pain, walking disability, facial nerve paresis, anisocoria, hydrocephalus, fatigue, aphasia, and dyslexia.

**Table 1:** Characteristics of children stratified by resection status

Characteristic	All	Primary	Recurrent	*p* value
*n*	78	45	33	
Age (years)				
± SD average	12.0 ± 4.39	12.3 ± 4.67	11.7 ± 3.94	*t* test: 0.50
Range	1.6–17	4–17	1.6–18	
Median	13	14	12	
Sex				
Female (*n*)	27	14	13	Chi2: 0.48
Male (*n*)	51	31	20	
KPS				
Median	90	90	90	
Range	30–100	60–100	30–100	Chi2: 0.89
<=60 *n* (%)	12 (15.4)	6 (13.6)	6 (17.6)	
70–80	17 (21.8)	10 (22.7)	7 (20.6)	
90–100	49 (62.8)	28 (63.6)	21 (61.8)	
Weight (kg)				
Avg ± SD	44.2 ± 19.4	45.2 ± 20.5	42.8 ± 17.8	*t* test: 0.61
Median	42.4	42.5	42.4	
Range	11–95	11.9–82	11–82

**Fig. 2:**
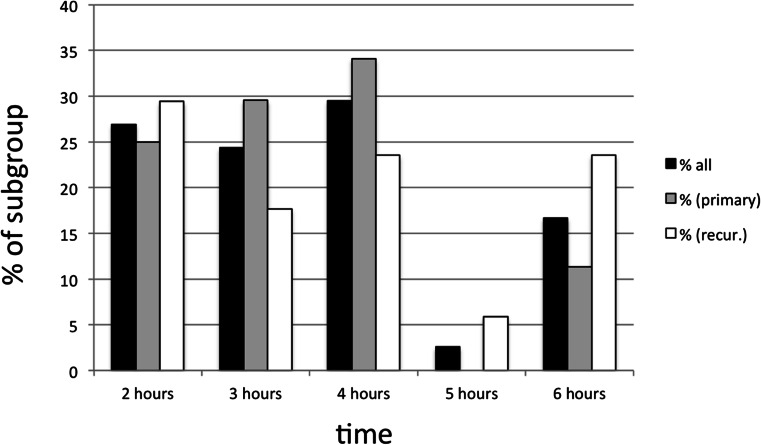
Distribution of administration time of complete cohort, stratified by recurrence status

All children were given 20 mg/kg Gliolan orally dissolved in tap water. However, there was a wide variation in timing of application of 5-ALA solution prior to surgery, as detailed in Fig. 2. Solutions were generally administered between 2 and 4 h prior to surgery. A distinct group of children was given solution at 6 h prior to surgery.

Apart from fluorescence, other methods for location of tumor were used intra-operatively, with neuronavigation employed in 57 of 78 (73.1 %) cases, ultrasound in 35 of 78 (44.9 %) cases, and intra-operative CT in one case. Mapping/monitoring techniques were used in 17 of 78 children (21.8 %).

**Fig. 3:**
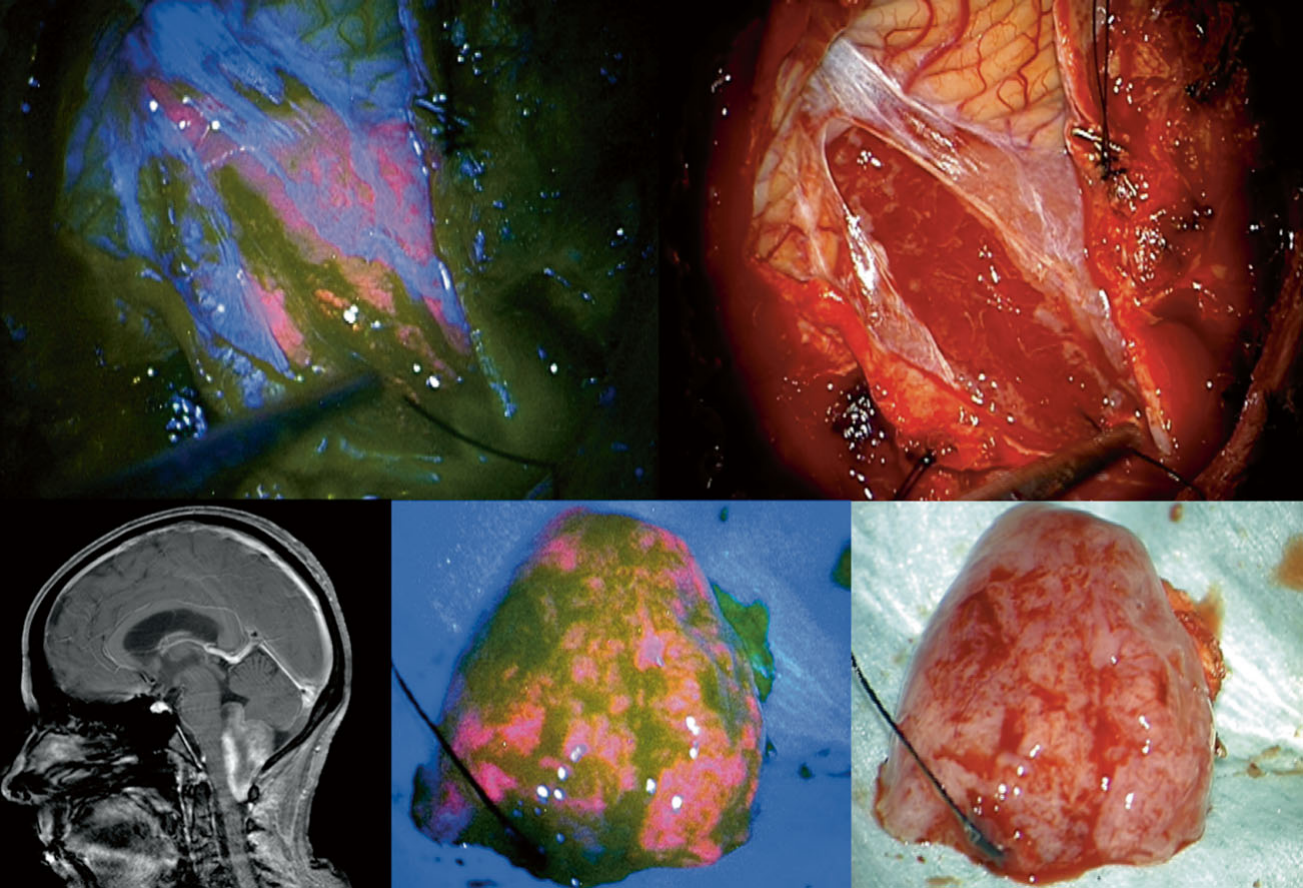
Example of fluorescence in ependymoma grade II (female, 12 years of age). *Top left*: blue light image of typical red fluorescence characterized as strong and related to ependymoma tissue (Zeiss Pentero); *top right*: Corresponding white light image. *Bottom left*: preoperative MRI showing tumor of 4th ventricle with patchy contrast-enhancement; *middle*: blue light image of tissue specimen; *right*: corresponding white light image

As expected, the variability of different tumors that were considered amenable to fluorescence-guided resection prior to surgery was high. The most frequent tumors were glioblastomas, followed by anaplastic astrocytomas, ependymomas grade III, PNETs, gangliogliomas, medulloblastomas, and pilocytic astrocytomas. These seven entities accounted for 59 of 78 tumors (>4 patients, Table 2). All of these tumors showed contrast enhancement to a variable extent on MRI. Fluorescence, however, was inconstant and was found to be useful in only 28 of these patients, the least commonly in medulloblastomas and pilocytic astrocytomas and inconsistently in gangliogliomas and PNETs. Among the infrequent entities found in this series (<4 patients, Table 3), all three ependymomas grade II showed useful fluorescence (Fig. 3). It was notable that the only meningiomas in this group also revealed useful fluorescence, as well as did a meningeal sarcoma.

**Table 2:** Pre-operative contrast enhancement, intra-operative fluorescence characteristics and “usefulness” of fluorescence in tumors with a frequency >4 in this cohort

Subgroup	*n*	First surgery	Recurrence	Strong c+	Weak/ patchy c+	No c+	Supratentorial	Infratentorial	Strong flu.	Weak/patchy flu.	No flu.	Useful flu.
GBM *n* (%)	14 (100.0)	8 (57.1)	6 (42.9)	13 (92.9)	1 (7.1)	0 (0.0)	13 (92.9)	1 (7.1)	10 (71.4)	3 (21.4)	1 (7.1	12 (85.7)
AA *n* (%)	5 (100.0)	5 (100.0)	0 (0.0)	2 (40.0)	3 (60.0)	0 (0.0)	5 (100.0)	0 (0.0)	2 (40.0)	2 (40.0)	0 (0.0)	3 (60.0)
Ependymoma III *n* (%)	7 (100.0)	2 (33.3)	5 (2.0)	4 (66.7)	3 (33.3)	0 (0.0)	4 (66.7)	3 (33.3)	4 (50.0)	1 (16.7)	2 (33.3)*	4 (50.0)
PNET *n* (%)	7 (100.0)	1 (14.3)	6 (85.7)	2 (28.6)	5 (71.4)	0 (0.0)	7 (100.0)	0 (0.0)	1 (14.3)	2 (28.6)	4 (57.1)	3 (42.9)
Ganglioglioma *n* (%)	5 (100.0)	4 (80.0)	1 (20.0)	2 (40.0)	3 (60.0)	0 (0.0)	5 (100.0)	0 (0.0)	0 (0.0)	2 (40.0)	3 (60.0)	2 (40.0)
Medulloblastoma *n* (%)	8 (100.0)	6 (75.0)	2 (25.0)	2 (25.0)	5 (62.5 %)	1 (12.5)	0 (0.0)	8 (100)	0 (0.0)	4 (50.0)	4 (50.0)	2 (25.0)
PA *n* (%)	13 (100.0)	9 (69.2)	4 (30.8)	11 (84.6)	2 (15.4)	0 (0.0)	5 (38.5)	8**(61.5)	2 (15.4)	1 (7.7)	10 (76.9)	2 (15.4)

Abbreviations: c+: contrast enhancement on MRI; flu.: visible fluorescence

*One child vomited immediately after ingestion

**One tumor with spinal cervical intramedullary location

**Table 3:** Pre-operative contrast enhancement, intra-operative fluorescence characteristics, and “usefulness” of fluorescence in tumors with a frequency <4 in this cohort

Subgroup	*n*	First surgery	Recurrence	Strong c+	Weak/ patchy c+	No c+	Supratentorial	Infratentorial	Strong flu	Weak/patchy flu	No flu	Useful flu *n* (%)
Ependymoma II	3	0	3	3	0	0	2	1	2	1	0	3 (100)
Oligodendroglioma II	2	2	0	0	2	0	2	0	1	0	1	1 (50)
Oligodendroglioma III	2	1	1	2	0	0	2	0	0	1	1	0 (0)
DNET	2	2	0	1	1	0	2	0	1	0	1	1 (50)
PXA II	2	1	1	1	0	1	2	0	0	0	2	0 (0)
DA II	1	1	0	0	1	0	1	0	0	1	0	0 (0)
Glioneural tumor IVth ventricle	1	1	0	0	1	0	0	1	0	1	0	1 (100)
Plexus papilloma II	1	0	1	0	1	0	1	0	0	0	1	0 (0)
Papillary meningiomas	1	0	1	1	0	0	1	0	1	0	0	1 (100)
Lipoma	1	1	0	0	1	0	0	1*	0	0	1	0 (0)
Neuroblastoma metastasis	1	1	0	0	1	0	1	0	0	0	0	0 (0)
Meningeal sarcoma	1	1	0	1	0	0	1	0	1	0	0	1 (100)
Gliotic tissue (after medulloblastoma surgery)	1	0	1	0	1	0	0	1	0	1	0	0 (0)

Abbreviations: c+: contrast enhancement on MRI; flu.: visible fluorescence

*Spinal intramedullary location

For practical use, it would be of value to determine factors *ex ante* for predicting useful fluorescence before applying 5-ALA. To identify such factors, single and multiple factor nominal-logistic regression analysis was performed (Table 4). For this analysis, two children with spinal tumors (one lipoma, one pilocytic astrocytoma) were omitted. Neither a single nor a combination of multiple factors was found to significantly predict the accumulation of “useful” fluorescence. However, there was a tendency for location (supra-, infratentorial) recurrence status (recurrence yes/no) and contrast enhancement on the pre-operative MRI to predict “usefulness”.

**Table 4:** Multivariate regression analysis of *ex ante* factors for predicting useful fluorescence in 76 cases (spinal tumors were omitted); “useful” was defined as a change of surgical strategy or detection of residual tumor from visible fluorescence. For multivariate analysis, all covariates were included with a univariate *p* < 0.4

Characteristic	*n*	Useful *n* (%)	Univariate *p*	Multivariate *p*
	76	36 (47.4)		
Contrast enhancement				
None	3	1 (33.3)	0.333	0.5653
Weak/patchy	29	11 (37.9)		
Strong	44	24 (54.4)		
Recurrence				
Yes	34	18 (52.9)	0.381	0.6193
No	42	18 (42.9)		
Location				
Supratentorial	53	28 (52.8)	0.125	0.2202
Cerebellar	23	8 (34.8)		
Gender				
Male	50	24 (48)	0.761	-
Female	26	12 (46.2)		
KPS				
<80	10	8 (80)	0.0637	0.821
70–80	12	6 (50)		
90–100	54	22 (40.7)		
Age (years)				
<8	19	36.8	0.761	-
9–13	23	52.2		
14–16	18	50		
>16	16	50		

Two spinal cases omitted (spinal lipoma and spinal pilocytic astrocytoma)

**Fig. 4:**
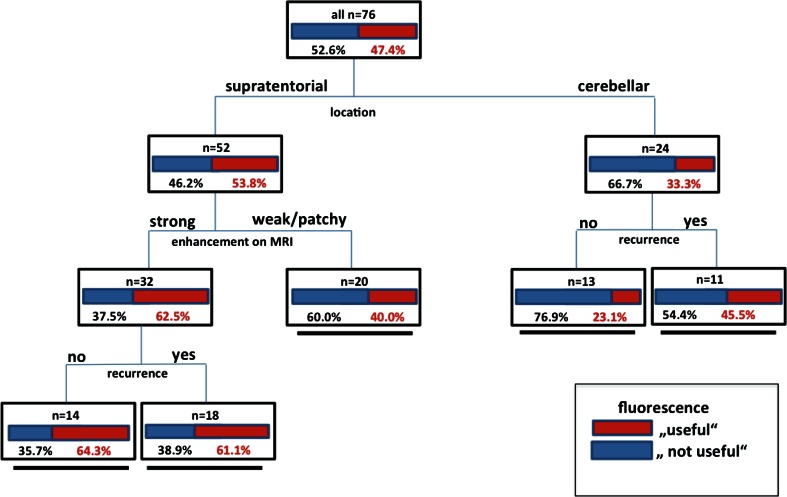
Decision tree generated from recursive partitioning analysis for *ex ante* determination of the likelihood for “usefulness”. Factors: Location; contrast enhancement on MRI, recurrence status (“useful” = provoking a change in surgical strategy or helping detect residual tumor; two children with spinal tumors excluded). There are five terminal nodes based on the likelihood of “usefulness” of fluorescence after three splits

Recursive partition analysis (RPA) was employed using these factors in order to better understand the interaction between these factors and the criterion “usefulness”. RPA generated a decision tree with five terminal nodes after three splits with different groups of patients characterized by varying likelihoods for the “usefulness” of fluorescence. Usefulness was highest in patients with tumors located supratentorially, strong contrast enhancement on MRI, and first surgery (64.3 %) as compared to patients with infratentorial tumors and first surgery (23.1 %, Fig. 4).

“Complete” resections on post-operative MRI were documented in 62 patients (79.5 %; one missing). However, there was no significant relationship between fluorescence manifestation, usefulness of fluorescence, and completeness of resection.

Table 5 summarizes adverse events reported in this study. Study participants reported no toxicological adverse events and in the majority of cases, no neurological adverse events were given. However, adverse events were recorded in eight of 78 patients (Table 5). In four of these patients, either weak/patchy or strong fluorescence was found, resulting in “useful” fluorescence in only two patients, one with post-operative hygroma after resection of a grade II ependymoma, and one with transient hoarseness after resection of a grade III ependymoma of the posterior fossa. In one patient (recurrence for medulloblastoma), unspecific fluorescence was found in gliotic tissue. Unspecific fluorescence was also described in one patient with recurrent oligodendroglioma grade III. In both cases, fluorescence was characterized as weak/patchy.

**Table 5:** Listing of reported adverse events with histology and fluorescence findings

Age	Final histology	WHO grade	Recurrence	Tumor location	Adverse events/complications
6	Pilocytic astrocytoma	I	No	Hypothalamic-chiasmatic	Cerebral salt wasting, vasospasm with hemiparesis right side
15	Glial fibrillary astrocytoma	II	No	Supratentorial	Resolution of neurological impairment
7	Medulloblastoma	IV	No	Cerebellar	Transient posterior fossa syndrome
12	Pilocytic astrocytoma	I	Yes	Supratentorial	Complex seizures/absences within first week post surgery
12	Ependymoma	IV	Yes	Cerebellar	Cerebellar neurological deficits
12	Ependymoma	III	Yes	Cerebellar	Transient hoarseness
16	Ependymoma	II	Yes	Supratentorial	Hygroma
11	Anaplastic oligodendroglioma	III	Yes	Supratentorial	Pre existing shunt became infected and dysfunctional

## Discussion

Gliolan (5-ALA) is approved for fluorescence-guided resections of malignant gliomas in Europe and other countries in adults but not in children. Thus, any use in children is strictly off-label. In this patient population, a general recommendation for the use of 5-ALA cannot be given and thus should be restricted to selected cases, since the safety profile has not been established, especially in young children. Nevertheless, there is now indication from the literature that 5-ALA appears to be used in several centers on an off-label basis despite the absence of data from controlled studies, especially on safety. In addition, the methods of application are not standardized and even in the present series the timing of administration varied widely. This is an undesirable development. Obviously, controlled studies are needed to determine both whether 5-ALA is toxicologically safe in children and whether 5-ALA is useful for facilitating detection or visualization of typical pediatric brain tumors.

In contrast to adults, the spectrum of brain tumor types is much broader in children. Not all tumor types may be good candidates for 5-ALA fluorescence-guided resections, even when they are intra-axial and show contrast enhancement on MRI. Especially pilocytic astrocytomas of the posterior fossa may be strongly enhancing and in some cases may be confused for medulloblastomas from imaging alone. Since they are benign in nature and usually well circumscribed intra-operatively, they may not be suitable for fluorescence-guided resections.

Schwake et al. [26] have demonstrated that cultures of cells derived from childhood brain tumors show differences in vitro regarding the level and the time course of PpIX accumulation. Medulloblastoma, PNET, and malignant glioma cell lines all showed variable results. Similar differential accumulation has been observed in medulloblastoma cell lines by others [27]. These observations alone question the expectation that pediatric brain tumors, even if malignant, will uniformly show useful fluorescence.

Thus, designing a study for the use of 5-ALA in children requires careful planning regarding which types of potential tumors to include. Since the available data collected and published so far are restricted, we set out to compile cases of children treated by European Gliolan users certified in the context of the Risk Management Plan imposed by EMA.

The resulting series is the largest to date with a collection of 78 children, now allowing more distinct conclusions regarding the usefulness of 5-ALA in this patient population, when 5-ALA is used in the same dosage and with comparable timing as compared to the adult population.

The results show glioblastomas and anaplastic astrocytomas to be similarly amenable to fluorescence-guided resections as in adults. It was interesting to determine that nine of ten ependymomas, regardless of whether they were grade II or III, showed fluorescence, and in eight of these cases fluorescence was considered useful for resection (Tables 2 and 3). Notably, the only child without intra-operative tumor fluorescence regurgitated the 5-ALA solution immediately after ingestion. If ependymoma is suspected, 5-ALA may be a good technical adjunct to surgery.

On the other hand, pilocytic astrocytomas only showed useful fluorescence in a minority of cases. Surprisingly, medulloblastomas, which are considered malignant, revealed useful fluorescence in only one-quarter of the eight available cases.

Although being only of academic value at this stage, the single meningiomas and meningeal sarcoma displayed useful fluorescence. Useful fluorescence in meningiomas resection has been described in several adult series [11, 13]. These observations show that “malignancy” or contrast enhancement by themselves do not represent independent factors that will reliably predict the accumulation of “useful” fluorescence in pediatric brain tumors. Also, since so far the mode of application of 5-ALA used in this series is similar to adults, it remains to be elucidated whether different dosages or timing will be more adequate for tumors that showed inconsistent fluorescence accumulation in this series, e.g., gangliogliomas or PNETs.

From a practical point of view, a histological diagnosis is generally not available prior to definite surgery (with the exception of previous biopsy). Thus, algorithms are necessary that allow *ex ante* predictions of the availability of useful fluorescence. From our analysis, we propose a decision tree based on recursive partition analysis. This analysis shows children with supratentorial, strongly contrast-enhancing tumors to have the greatest potential benefit from 5-ALA, if histology is unknown and if the timing and dosages that have been found useful in the adult malignant glioma population are employed.

In two patients, the occurrence of unspecific fluorescence in single samples was noted, always weak in nature and confined to gliosis. Although fluorescence is considered highly predictive in recurrent gliomas with a positive predictive value of over 95 % [28], weak fluorescence has sometimes been found in these patients and related to tissues to gliosis and scarring but not to functional brain. Similar tissue alterations cannot principally be ruled out in recurrent pediatric brain tumors.

Reported complications in this series were few and not evidently related to the use of 5-ALA, although a standardized analysis of laboratory values has not been performed. In their retrospective series of 16 children treated with 5-ALA, Beez et al. [25] reported a transient increase in liver enzymes, which has also been reported in adults [29]. In adults, this elevation is transient and has not been considered an indicator of liver damage but rather related to metabolism of 5-ALA and its downstream metabolites.

Given the present data, a future trial designed by our group in children will only consider intraaxial, strongly contrast-enhancing supratentorial tumors either in the primary or in the recurrent setting. We justify the exclusion of children with posterior fossa tumors in a context of first controlled trial by the low frequency of useful fluorescence observed in this group, especially in a first surgery setting without previous histological diagnosis. In these cases, less than 25 % of children revealed tumors with useful fluorescence due to the high incidence of pilocytic astrocytomas in patients with first surgery. This trial will primarily focus on toxicological safety and secondarily on the positive predictive value of fluorescence for detecting and visualizing tumor. The timing of administration will be uniform and earlier than found in the present study, given experimental data available so far for in vitro childhood brain tumors [26].

Such a study does not rule out the future use of 5-ALA in entities in which the accumulation of useful fluorescence is less likely, as suggested by the current data, but only on the basis of established safety data.

A future randomized study in pediatric patients with a particular tumor subtype, e.g., glioblastoma with survival as an endpoint, is unlikely to be designed due to the overall paucity of these tumors and the established use of 5-ALA in adult patients.
